# Data on greenhouse gases emission in condensate separation unit of a petrochemical company in Iran

**DOI:** 10.1016/j.dib.2016.06.041

**Published:** 2016-06-29

**Authors:** Mehdi Ahmadi, Mehrshad Dastorian, Nemat Jafarzadeh, Sahand Jorfi, Bahman Ramavandi

**Affiliations:** a Environmental Technologies Research Center, Ahvaz Jundishapur University of Medical Sciences, Ahvaz, Iran; b Department of Environmental Health Engineering, Ahvaz Jundishapur University of Medical Sciences, Ahvaz, Iran; c Department of Chemical Engineering, Azad University of Mahshahr, Iran; d Department of Environmental Health Engineering, Bushehr University of Medical Sciences, Bushehr, Iran

**Keywords:** Greenhouse gas emissions, Emission factor, Petrochemical, Clean Development Mechanism, Bandar Imam Petrochemical Complex

## Abstract

Since global warming due to greenhouse gas emissions is no respecter of geographical boundaries of countries, concerted mitigation activities such as Clean Development Mechanism (CDM), are suitable. In this mechanism, some developed countries can gain certified emission reduction credits from emission reduction actions undertaken in developing countries. Thus, the data of greenhouse gas emissions in developing countries would be informative for implementing of CDM. Herein, the data of greenhouse gas emissions of Bandar Imam Petrochemical Complex, one of the biggest petrochemical companies in the Middle East region is presented. The data was acquired using emission factor method and self-presented raw information of the Bandar Imam Petrochemical Complex. Overall, the data will be interesting for environmentalists, non-governmental organization (NGO), and developed countries to perform CDM.

**Specifications Table**

**Table:** 

Subject area	*Environmental Engineering*
More specific subject area	*Air Pollution*
Type of data	*Table and image*
How data was acquired	Collect raw data of greenhouse gas emission from an Iranian petrochemical company.
	Use emission factor to calculate greenhouse gases
*Data format*	*Processed, raw*
Experimental factors	*Processing of greenhouse gas emission data*
Experimental features	*Contribution of condensate separation unit of a Petrochemical Plant in Iran in greenhouse gas emission*
Data source location	*Mahshahr, Iran, 30°33′32″N 49°11′53″E*
Data accessibility	*Data is available with the article*

**Value of the data**

• This data set generally answered the question of “what is the situation of the implementation of Kyoto protocol legislations to prevent/reduce greenhouse gas emissions in companies in developing countries such as Bandar Imam Petrochemical Complex (BIPC)?”
• The data will be attractive for whom with concern about global warming such as non-governmental organization (NGO).
• The data of greenhouse gases estimation by emission factor in this article implicitly proposes that Bandar Imam Petrochemical Complex is good place for carbon trade and Clean Development Mechanism (CDM) implementation.

## 1 Data

**Fig. 1:**
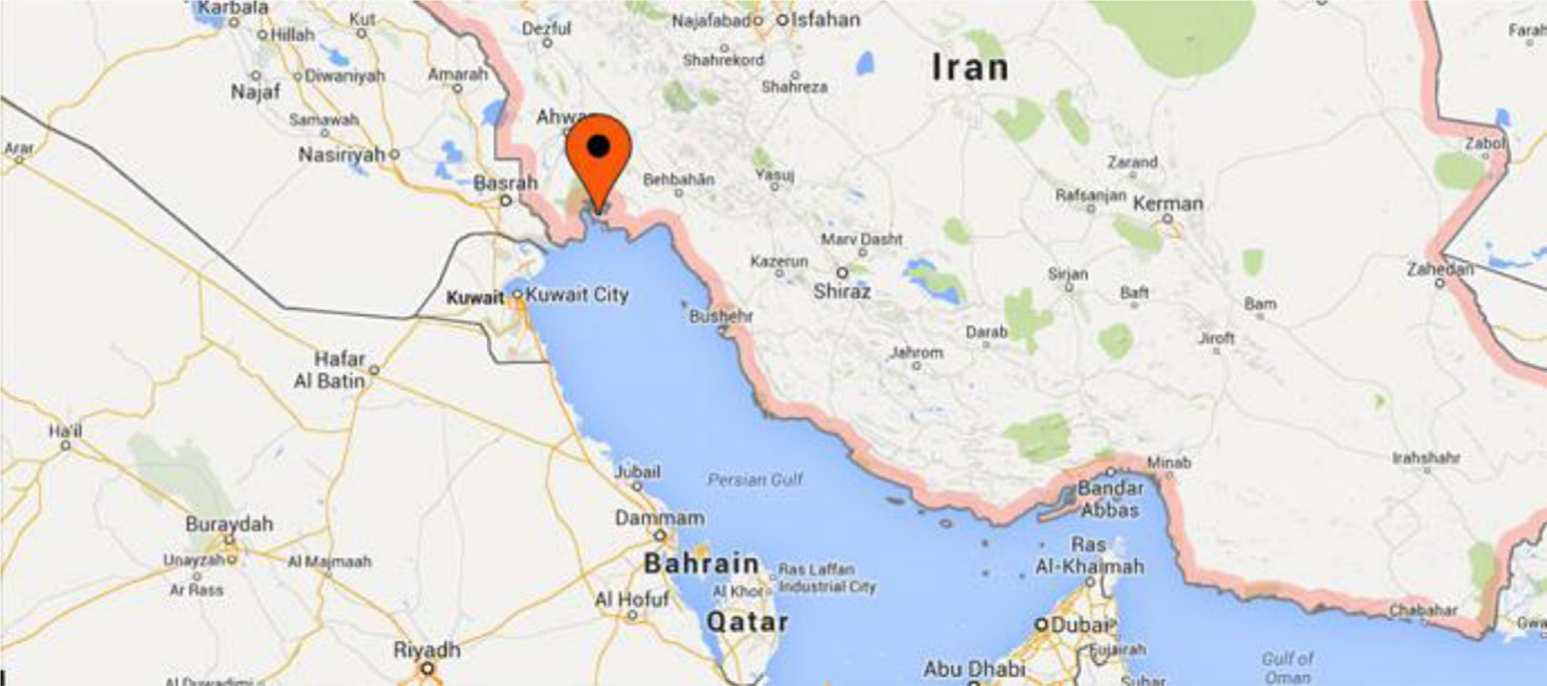
Geographical map of the site study.

Data presented here describe the greenhouse gases especially CH_4_ and CO_2_ emission from a petrochemical plant with condensate separation unit in Mahshahr, Iran. Two Tables and one figure are presented. Fig. 1 is depicts the geographical position of the Bandar Imam Petrochemical Complex (study zone). Table 1 shows emission of CH_4_ and CO_2_ and Table 2 contains the emission factors presented by different references.

## 2 Experimental design, materials and methods

The data of this article was obtained from Bandar Imam Petrochemical Complex (BIPC), with an area of 270 ha, which is located in the North West coast of the Persian Gulf. This petrochemical company is situated in Khuzestan province, Iran with 105 km southeast of Ahvaz city and 84 km East of Abadan and Mahshahr cities (see Fig. 1).

The estimation process of greenhouse gases emission involved three stages: In the first stage, a site survey with process flow diagram (PFD) study was done in September 2015 for analyzing components attributed in greenhouse gases emission in unit of separating gas condensate of Bandar imam petrochemical company. In the second stage, the emission factors provided by various organizations, which have been listed in Table 1, emissions for each sources was calculated by using Eq. (1):

(1) E=A×EF×[1-(ER/100)]

In this equation, *E* is the rate emission of greenhouse gas (the amount of greenhouse gas mass); *A* is the amount of activity; EF is an emission factor (the amount of greenhouse gas mass emitted per the amount of product produced or the rate activity); ER is the overall percentage reduction of emission that this value is considered to be zero, due to the lack of using greenhouse gas reduction systems [1]. Finally, the data were processed using Excel software for calculation of emission rate by formulation Eq. (1).

## Figures and Tables

**Table 1:** CH_4_ and CO_2_ emission from condensate separation unit.

**Unit**	**Greenhouse emission**		**References**
	**CO**_**2**_**(Tonne/day)**	**CH**_**4**_**(Tonne/day)**	
Flare	–	0.25	[2]
	44.85	0.32	[3]
	–	0.05	[4]
	49.53	–	[5]
	57.85	–	[6]
	43.08	0.16	[7]
	61.12	0.005	[8]
	59.36	0.25	[9]
Gas Heater	–	0.001	[10]
	29.43	0.006	
	–	0.99	[3]
Reboiler	0.15	1.38×10^−6^	[11]
Separators	–	0.03	[12]
Compressor station	0.004	0.01	[10]
Compressor turn on	0.03	0.53	
Compressor blow down	0.10	0.24	
Propane Reservoir	4.23	1.81	[10]
Butane Reservoir	4.23	1.81	
Pentane Reservoir	1.88	0.80	
Hexane Reservoir	0.86	0.37	
Gas Valves	–	0.10	[10]
Natural Gas combustion	1718.05	–	[13]
	1683.19	–	[10]
	1683.19	0.03	[10]
	1725.38	0.03	[14]
	1686.54	–	[6]

**Table 2:** CH_4_ and CO_2_ emission factors.

**Unit**	**References**	**Emission factors**	
		**CO**_**2**_	**CH**_**4**_
Flare	[2]	–	0.61 lb/MMBtu
	[3]	1853 g/m^3^ gas	13.6 g/m^3^ gas
	[4]	–	0.12 lb/MMB
	[5]	120.72 lb/MMBtu	–
	[6]	141.01 lb/MMBtu	–
	[7]	105.01 lb/MMBtu	0.39 lb/MMBtu
	[8]	148.98 lb/MMBtu	0.01 lb/MMBtu
	[9]	144.69 lb/MMBtu	0.61 lb/MMBtu
Gas Heater	[10]	–	1.1×10^−^^6^ tonne/10^6^ Btu
	[3]	1891 g/m^3^	0.04 g/m^3^
	[12]	–	20.987 Scf /heater-yr
Re boiler	[11]	116.87 lb/MMscf	0.011 lb/MMscf
Separators	[12]	–	20171 scf/separator-yr
Compressor station	[10]	2.42*10^−2^ tones/vessel-yr	2.42×10^−2^ tones/vessel-yr
Compressor turn on		2.42*10^−2^ tones/vessel-yr	2.42×10^−2^ tones/vessel-yr
Compressor blow down		2.42*10^−2^ tones/vessel-yr	2.42×10^−^^2^ tones/vessel-yr
Gas Valves	[10]	–	4.5×10^−6^ tonne /hr/component
Heavy Oil valves		–	8.4×10^−9^ tonne /hr/component
Light Oil valves		–	2.5×10^−6^ tonne /hr/component
Valves Oil/Water		–	9.8×10^−8^ tonne /hr/component
Natural Gas combustion	[13]	0.05 tonne/MMBtu	–
	[10]	0.05 tonne/MMBtu	1.06×10^−6^ tonne/MMBtu
	[14]	120000 Lb/106scf	2.3 Lb/106scf
	[6]	0.05 tonne/MMBtu	–
